# BRCA1: An Endocrine and Metabolic Regulator

**DOI:** 10.3389/fendo.2022.844575

**Published:** 2022-03-31

**Authors:** Haim Werner

**Affiliations:** Department of Human Molecular Genetics and Biochemistry, Sackler School of Medicine, Tel Aviv University, Tel Aviv, Israel

**Keywords:** BRCA1, tumor suppressors, p53, insulin-like growth factor-1 (IGF1), estrogen receptor, transcription

## Abstract

The breast and ovarian cancer susceptibility gene (BRCA1) is a tumor suppressor whose mutation has been associated with the development of breast, ovarian and, probably, other malignancies at young ages. The BRCA1 gene product participates in multiple biological pathways including the DNA damage response, transcriptional control, cell growth and apoptosis. Inactivating germline mutations of the *BRCA1* gene can be detected in a substantial portion of families with inherited breast and/or ovarian cancer. While the genomic and cancer-related actions of BRCA1 have been extensively investigated, not much information exists regarding the cellular and circulating factors involved in regulation of *BRCA1* expression and action. The present review article dissects the emerging role of BRCA1 as an important regulator of various endocrine and metabolic axes. Experimental and clinical evidence links BRCA1 with a number of peptide and steroid hormones. Furthermore, comprehensive analyses identified complex interactions between the insulin/insulin-like growth factor-1 (IGF1) signaling axis and BRCA1. The correlation between metabolic disorders, including diabetes and the metabolic syndrome, and *BRCA1* mutations, are discussed in this article.

## Discovery and Early Characterization of BRCA1

The race for the identification of the gene responsible for inherited breast and ovarian cancer ended in 1994 with the cloning of the *BRCA1* gene by Miki and colleagues (1, 2). Positional cloning methodology allowed identification of a 17q-linked gene whose mutation affected susceptibility to breast and ovarian cancer. The *BRCA1* gene encodes a predicted protein of 1863 amino acids, containing a distinct ring finger element in its N-terminal domain. The high penetrance of the *BRCA1* gene was recognized early on in the course of *BRCA1* characterization by analyses showing that mutation carriers have an increased lifetime risk of developing breast (40–85%) and/or ovarian (16–64%) cancers (3–8). In classical terms, *BRCA1* fits the criteria of a candidate tumor suppressor gene and some of its archetypal biological activities are described in the next section.

While inactivating *BRCA1* germline mutations are linked to a small portion of the total number of breast tumor cases worldwide, the cloning and subsequent characterization of the *BRCA1* gene had an unprecedented impact on our understanding of breast cancer etiology (9). In fact, lessons learned from molecular and genetic analyses of *BRCA1* transcended the area of familial breast and ovarian cancer and are regarded as universal biological paradigms in cancer (10). In addition to its genomic and cancer-related activities, more recent evidence revealed that BRCA1 displays a number of metabolic and hormone-like types of action. The present review article focuses on the involvement of tumor suppressor BRCA1 in endocrine system control. Our assay attempts to shed new light on the rapidly expanding spectrum of actions of BRCA1. Their potential clinical ramifications are discussed in detail.

## BRCA1 Develops its Identity

**Figure 1:**
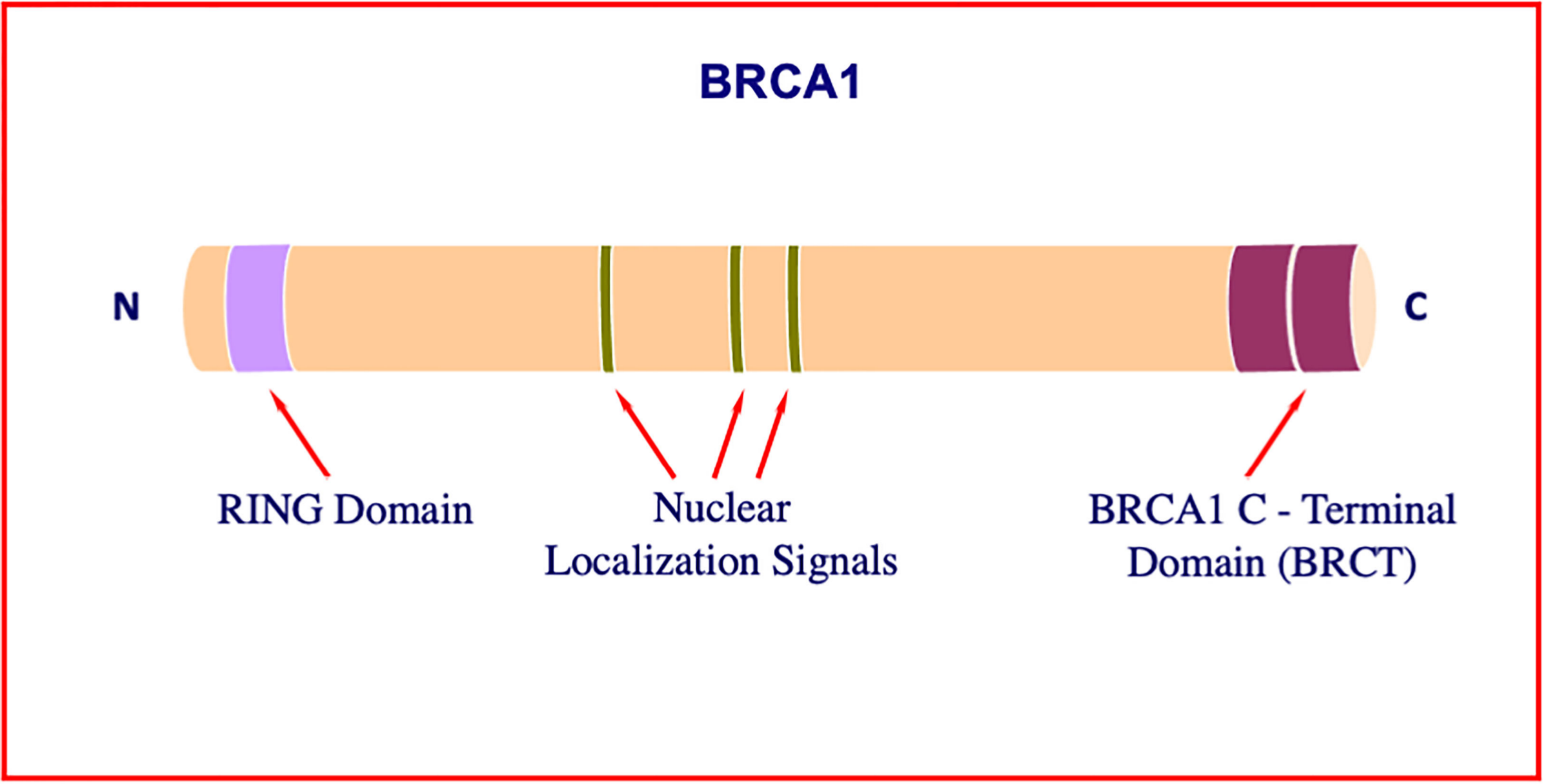
Structure of BRCA1. The *BRCA1* gene encodes a 1863-amino acid protein with tumor suppressor activity. BRCA1 plays a critical role in DNA damage sensing and it forms a complex that repairs double-strand breaks. The N-terminal portion of the molecule includes a particular type of zinc finger element, termed RING motif. Among other roles, this domain interacts with proteins involved in BRCA1 ubiquitination. The central portion of the molecule includes a number of nuclear localization signals. The tandem C-terminal BRCT domain has important roles in DNA repair, transcription regulation and tumor suppressive functions.

Early studies identified BRCA1 as a critical player in the maintenance of genomic stability (11–13). Consistent with this role, cells with a defective *BRCA1* gene exhibit a series of typical anomalies, including impaired DNA damage response, defects in homologous recombination with ensuing low efficiency DNA repair, and faulty cell cycle checkpoints (14–16). BRCA1 was shown to interact with a wide range of molecules, including BARD1 (*via* its N-terminal ring finger domain), DNA repair enzymes (mainly *via* its central domain), and transcriptional activators (primarily *via* two tandem BRCA1 C-terminal, or BRCT, motifs) (17–19) (**Figure 1**). A number of excellent review articles focusing on the physical and functional interactions of BRCA1 have been published (18, 20).

The involvement of BRCA1 in transcriptional regulation is supported by solid experimental evidence (21, 22). Specifically: (i) BRCA1 is predominantly found within the nucleus (23, 24), albeit DNA damage as well as viral infection can alter its subcellular distribution (25); (ii) BRCA1 has been identified as a component of the RNA polymerase II holoenzyme by a number of biochemical criteria (19); (iii) the C-terminal domain of BRCA1 is highly acidic and exhibits a potent transcriptional transactivation activity (21, 26); and (iv) the N-terminal ring finger element resembles a similar motif described in several DNA-binding proteins, including the Wilms’ tumor suppressor, WT1 (1). Finally, a novel transcriptional mechanism responsible for autoregulation of *BRCA1* gene transcription has been described (27). This regulatory loop involves the formation of a multimeric complex that contains, in addition to BRCA1, nuclear proteins E2F1 and RB. This complex displays a constitutive repressive activity pattern that leads to inhibition of *BRCA1* transcription. Disruption of the complex by various genotoxic stresses results in displacement of the BRCA1 protein from the *BRCA1* promoter region with subsequent upregulation of *BRCA1* transcription.

## BRCA1 Inhibits IGF1 Receptor Gene Expression and Action

The insulin-like growth factors (IGF1, IGF2) have an important role in the development and maturation of the mammary gland. In addition, IGFs are key players in breast cancer initiation and progression (28–31). Epidemiological analyses conducted over the past twenty-five years identified IGF1 as a risk factor for breast cancer (32–34). These population studies are in agreement with IGF1 function as a progression factor during the cell cycle (35, 36). Furthermore, studies reflect the well-established pro-survival role of IGF1 as well as its involvement in metabolic and nutritional control. The cell-surface IGF1 receptor (IGF1R), which mediates the biological actions of both IGF1 and IGF2, is regarded as a central player in breast cancer (31, 37–39). Constitutive activation of the IGF1R tyrosine kinase domain is a common event in cancer cells, although the prognostic significance of IGF1R levels and activation status in clinical settings remain unsettled (40).

**Figure 2:**
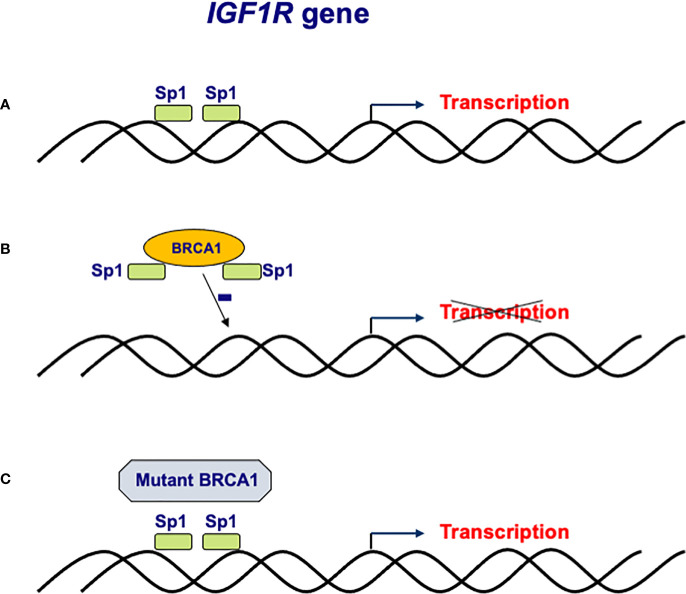
Regulation of *IGF1R* gene expression by BRCA1. **(A)** The *IGF1R* gene promoter includes a number of high affinity binding sites for transcription factor Sp1 in its proximal region. **(B)** The mechanism of action of BRCA1 involves binding to, and sequestration of, Sp1, thus preventing its binding to *cis-*elements in the promoter region. Lack of Sp1 binding leads to a reduction in *IGF1R* promoter activity and IGF1R levels. **(C)** *Loss-of-function* mutation of BRCA1 in familial and, potentially, sporadic breast cancer may lead to inactivation of BRCA1. Mutant BRCA1 is unable to bind Sp1 and suppress *IGF1R* gene transcription. Enhanced IGF1R levels are usually associated with augmented cell proliferation.

Molecular, genetic and biochemical analyses identified complex physical and functional interactions between BRCA1 and the IGF1 signaling pathway (41, 42). Consistent with its tumor suppressor role, wild-type BRCA1 was shown to repress *IGF1R* gene transcription and promoter activity as well as endogenous IGF1R levels in breast cancer cells (43, 44). In contrast, a truncated form of BRCA1 (185delAG, a mutation with a high incidence among Ashkenazi Jews) was unable to inhibit *IGF1R* promoter activity (45, 46). The mechanism of action of BRCA1 involves interaction with Sp1, a zinc-finger transactivator of the *IGF1R* gene (**Figure 2**). Specifically, binding of BRCA1 to Sp1 prevents Sp1 from binding the *IGF1R* promoter region, leading to reduction in IGF1R levels and ensuing decrease in IGF1-mediated proliferation. In agreement with the inability of mutant BRCA1 to suppress *IGF1R* transcription, primary breast tumors derived from *BRCA1* mutation carrier patients expressed significantly higher levels of IGF1R than sporadic breast tumors (47).

Of importance, the transcriptional activity of BRCA1 is strongly dependent on the cellular status of tumor suppressor p53. Thus, BRCA1 was capable of repressing *IGF1R* transcription in both p53-expressing and p53-null cellular backgrounds, but not in mutant p53-containing cells (48). As a corollary, *loss-of-function* mutation of the *p53* gene in human cancer may affect the capacity of BRCA1 to repress *IGF1R* gene expression, with major clinical implications (49). In addition to breast cancer, BRCA1 was identified as a transcriptional repressor of the *IGF1R* gene in prostate and endometrial cancer cells (50–53).

Besides the *IGF1R* gene, BRCA1 was shown to target other components of the insulin-IGF1 axis. Intratumoral IGF1 concentrations were elevated in tumors from *BRCA1* or *BRCA2* mutation carriers compared with matched sporadic tumors (54). In addition, BRCA1 was shown to interact with the insulin receptor substrate-1 (*IRS-1*) promoter and to inhibit its activity (55). This effect at the promoter level was associated with epigenetic modifications of histone H3 and H4, leading to a transcriptional repressive chromatin configuration. Consistent with this inhibitory role, *BRCA1*-deficient mammary tumor cells exhibited high levels of IRS-1. Furthermore, suppression of *IRS-1* using RNA interference markedly inhibited cell growth. **Table 1** summarizes key concepts on the interaction between BRCA1 and the IGF1 axis.

**Table 1:** Interactions between BRCA1, steroid hormones and the IGF1 signaling pathway.

The *IGF1R* gene is a *bona fide* downstream target for BRCA1 action
Wild-type, but not mutant, BRCA1 suppresses *IGF1R* promoter activity
IGF1R levels are higher in tumors from *BRCA1* mutation carriers than in sporadic tumors
IGF1 and IGF2 enhance *BRCA1* gene expression
IGF1 levels are upregulated in tumors from *BRCA1/BRCA2* mutation carriers
BRCA1 inhibits *IRS-1* promoter activity
BRCA1 inhibits the estradiol-inducible transcriptional activity of ERα
The stress hormone hydrocortisone represses *BRCA1* gene expression
The glucocorticoid receptor physically interacts with the *BRCA1* gene promoter

## Developmental and Hormonal Regulation of *BRCA1* Expression

Ontogenetic analysis of *BRCA1* gene expression in the normal mouse showed that BRCA1 is highly expressed in rapidly proliferating cells (56). In addition, *BRCA1* expression is induced by positive growth signals at the cell cycle point where cells become committed to replicate their DNA and undergo cell division (57, 58). Maximal *BRCA1* expression was detected during the pre-replicative (G_1_) phase of the cell cycle (59), and it was proved that BRCA1 is involved in the control of the G_1_-S and G_2_-M transition checkpoints (12, 16, 60). Finally, structure-function analyses have demonstrated that the tandem BRCT domain is essential in cell cycle checkpoint control by interacting in a phosphorylation-dependent fashion with specific DNA damage-induced proteins (61).

While BRCA1 has an important role in regulating *IGF1R* gene expression, as described in the previous section, experimental evidence indicates that both IGF1 and IGF2 stimulate *BRCA1* expression in a dose-dependent fashion (62). The effect of the growth factors was mediated at the transcriptional level, as revealed by transfection experiments using *BRCA1* promoter-luciferase reporter constructs. Given the fact that IGFs regulate cell division by controlling events that occur mainly during G_1_, it is reasonable to assume that at least part of the bioactivities of the IGFs are mediated by BRCA1. This concept is supported by experiments showing that BRCA1 silencing was associated with a two-fold increase in the IGF1-induced portion of cells that arrested at SubG0, and with a ~33% reduction in the portion of cells at the M-phase. In view of the fact that IGF1 is mainly produced by stromal cells whereas IGF2 biosynthesis occurs directly in breast tumor cells, data indicate that *BRCA1* gene expression is potentially regulated by both autocrine (IGF2) and paracrine/endocrine (IGF1) stimuli (54).

Finally, AKT, a downstream IGF1 target, was shown to regulate BRCA1 stability independent of new protein synthesis (63). Hence, IGF1 signaling is capable of modulating BRCA1 abundance at various levels of regulation. Taken together, studies suggest that a feedback loop controls expression and action of the IGF1 and BRCA1 signaling pathways in a synchronized manner. Deregulated expression of *BRCA1* as a result of aberrant IGF signaling might bear consequences in breast cancer development (41).

## Metabolic Roles of BRCA1

The impact of obesity and diabetes on cancer risk in *BRCA1* mutation carriers has been the topic of major clinical concern (64–66). While obesity and hyperinsulinemia are well established risk factors for breast cancer, and given the fact that BRCA1 exhibits a number of metabolic types of action, it is of medical relevance to explore the effects of a defective BRCA1 pathway on the linkage between diabetes and breast pathologies. The BRCA1-induced metabolic reprogramming of breast cancer cells was examined using global metabolomics and transcriptomics platforms (67). Wild-type BRCA1 induced numerous metabolic modifications, including a marked inhibition of glycolysis. Thus, all glycolysis indicators were largely (~50%) decreased in BRCA1wild-type, in comparison to BRCA1 mutant, cells. Five major enzymes of this pathway, including HK2 and PFKFB3, and both pyruvate and lactate were down-regulated by BRCA1 transfection. On the other hand, the tricarboxylic acid (TCA) cycle and oxidative phosphorylation were activated in BRCA1-expressing cells. In addition, BRCA1 induced a decrease of ketone bodies and free fatty acids, which were probably employed to supply Acetyl-CoA for the TCA cycle. Furthermore, BRCA1-transfected cells displayed enhanced activity of antioxidative pathways, most likely as a result of ROS production by oxidative phosphorylation. The overall implication of these analyses is that BRCA1 is capable or reversing the Warburg effect (see **Table 2**). The impact of this novel mechanism on tumor suppression has yet to be assessed.

**Table 2:** Metabolic actions of BRCA1.

BRCA1 induces several metabolic modifications, including inhibition of glycolysis
BRCA1 activates the TCA cycle and oxidative phosphorylation
BRCA1 induces a decrease of ketone bodies and free fatty acids
BRCA1-transfected cells display enhanced activity of antioxidative pathways
Mutant BRCA1 leads to increased lipogenesis
BRCA1 depletion leads to reduced mitochondrial respiration and reduced ATP levels

A recent study examined the impact of reduced BRCA1 expression on metabolic reprogramming of ovarian cancer cells (68). Authors showed that BRCA1 depletion led to diminished mitochondrial respiration and reduced ATP concentrations. Of interest, these metabolic alterations sensitized the cells to agents that inhibit mitochondrial activity and glucose import. Hence, inhibition of energy metabolism might constitute a useful strategy to target BRCA1-deficient high grade serous ovarian cancer, a type of tumor characterized by frequent BRCA1 loss (69).

Bordeleau et al. examined the medical histories of 6,052 women with *BRCA1* or *BRCA2* mutations, half of whom had been diagnosed with breast cancer (70). Authors reported that there was no excess of diabetes among patients with breast cancer in the period before diagnosis, compared with control individuals without cancer. However, there was a doubling in the risk of diabetes among *BRCA1* or *BRCA2* mutation carriers in the 15-year period after diagnosis of breast cancer. Importantly, the risk was even higher for women with a BMI higher than 25. Authors suggested that the enhanced risk might be linked to weight gain after tumor therapy. Finally, Oliverio et al. examined the risk of metabolic exposures with respect to a number of BRCA1/2 variants in a cohort of 438 women carriers of *BRCA1/2* mutations (71). Authors reported that *loss-of-function* variant carriers had significantly higher levels of plasma glucose and serum insulin than nonsynonymous variant carriers. Given that *BRCA* mutations confer a lower ability to repair DNA damage, authors suggested that mutation carriers may be more sensitive to the proliferative effects of insulin.

## Involvement of BRCA1 in Lipogenesis

Given the connection between obesity and cancer risk, as described above, studies investigated the potential role of BRCA1 in regulation of lipogenesis and energy metabolism. Mutant *BRCA1* has been associated with increased lipogenesis due to relaxation of the repressive action of wild-type BRCA1 on acetyl-CoA carboxylase, a key enzyme in fatty acid synthesis (72). Furthermore, *BRCA1* mutation carriers seem to have decreased blood IGF-binding proteins concentrations and, sometimes, lack an allele containing cytosine–adenine repeats in the *IGF1* gene promoter, which has been linked to decreased insulin sensitivity (7).

A recent study by Koobotse et al. reported that loss of BRCA1 in breast cancer cells led to downregulation of a phosphorylated and inactive form of acetyl CoA carboxylase-α (ACCA) (73). This effect was linked to a concomitant increase in levels of fatty acid synthase (FASN). In addition, IGF1 stimulated de-phosphorylation of ACCA by inhibiting the interaction between BRCA1 and phospho-ACCA. In consequence, deficit of BRCA1 increased the non-genomic effects of IGF1 as well as the mitogenic response of cells to IGF1. Furthermore, the effect of high glucose, as compared to physiological concentrations, on the tumor suppressive role of BRCA1 was investigated (74). Normal glucose levels blocked ACCA dephosphorylation by enhancing the association between BRCA1 and phospho-ACCA. The mitogenic response of breast cancer cells to IGF1 was decreased under physiological glucose values whereas no differences were seen in normal mammary epithelial cells. Hence, it is reasonable to assume that normal glucose concentrations facilitate the role of BRCA1 as a metabolic restraint of IGF1 actions. As a corollary, maintaining physiological levels of glucose may improve BRCA1 function and delay breast cancer progression.

In conclusion, the association between metabolic disorders, including diabetes and the metabolic syndrome, and *BRCA1* and *BRCA2* mutations is of major clinical relevance and warrants further investigation.

## Interactions Between BRCA1 and Steroid Hormones

Early studies have identified functional interactions between BRCA1 and a number of steroid hormones, including the estrogen receptor-α (ERα) and androgen receptor (AR). BRCA1 inhibited the estradiol-inducible transcriptional activity of ERα in breast and prostate cancer cells whereas cancer-associated *BRCA1*-mutant cells did not exhibit depressed ERα activity (75, 76). On the other hand, estrogens are capable of enhancing *BRCA1* expression, probably as a result of the mitogenic activity of estrogens. In addition, a direct effect of estrogens was suggested by studies showing that estradiol directly stimulates *BRCA1* promoter activity.

Rosen et al. suggested that BRCA1 regulation of ERα signaling might be important in sporadic carcinogenesis given that this type of breast cancer, unlike *BRCA1*-associated tumors, are usually ERα-positive and often exhibit loss of BRCA1 expression. Loss of BRCA1 could result in unopposed estrogen stimulation of mammary epithelial cell proliferation (77). The impact of *BRCA1/2* mutations on steroid hormone activity was assessed by examining endometrial thickness for each menstrual cycle day as an index of hormone regulation in a cohort of 228 women in the UK Familial Ovarian Cancer Screening Study (78). In addition, estradiol and progesterone titers for the same days were measured. Authors reported that *BRCA1/2* mutation carriers were exposed to enhanced levels of both steroid hormones. Higher values of estradiol in mutation carriers are consistent with a potential carcinogenic role of this hormone in the ovary.

Evidence for functional interactions between BRCA1 and androgens was suggested by experiments showing differential regulation of the *IGF1R* gene by BRCA1 in androgen receptor (AR) positive, as compared to AR negative, prostate cancer cells (50). BRCA1 was expressed at relatively high levels in prostate cancer compared with a low BRCA1 immunostaining in normal prostate epithelium. In addition, there was a negative correlation between IGF1R and BRCA1 expression levels in AR-negative prostate cancer cells whereas in cells with an active AR there was a positive correlation. Finally, cotransfection experiments revealed that BRCA1 expression enhanced AR transcriptional activity. Taken together, analyses identified a new mechanism for IGF1R and AR stimulation of prostate cancer, and further support the relevance of targeting AR and IGF1R in this type of tumors with BRCA1 serving as a marker for defining the target activity.

## *BRCA1* Mutations and Reproduction

The potential impact of *BRCA1* mutations on reproduction and fertility has been the focus of major interest (79). However, despite many efforts involved data remain controversial. A study by Kwiatkowski et al. suggested that *BRCA1* mutations increase fertility in families at hereditary breast/ovarian cancer risk (80). Authors evaluated the following hypothesis: if mutations that favor cancer development have survived selection pressure through generations, it is reasonable to assume that these mutations must provide clear advantages that compensate for the reduction in life expectancy. Analyses were conducted on 2,150 families with hereditary cancer, including approximately 96,000 individuals. Authors reported that fertility advantages were seen in a subgroup of 746 *BRCA1* mutation carriers and 483 non-carriers from *BRCA1* mutated families. Female carriers were less often nulliparous (9.1% of carriers in comparison to 16.0% of non-carriers) and had more children (1.8 ± 1.4 *vs* 1.5 ± 1.3). Likewise, male carriers had more children (1.7 ± 1.3 *vs* 1.4 ± 1.3). While moderate, this increase in fertility in both male and female carriers is suggestive of a mechanism that compensates for shortening of the reproductive phase of life.

An additional report based on two longitudinal studies provides evidence that female *BRCA1/*2 mutation carriers had more children, shorter birth intervals and reproduced later in life when compared to matched controls (81). Authors suggested that the positive correlation between *BRCA1/2* mutations and fertility can probably be explained by reported associations between *BRCA* mutations and telomere length and between telomere length and fertility.

In contrast, a negative impact of *BRCA1/2* mutations on fertility was suggested by studies showing that these mutations negatively affect ovarian reserve through accumulated DNA damage (82). Ovarian stimulation was performed in 126 women with breast cancer by using letrozole and gonadotropins for the purpose of fertility preservation by embryo or oocyte cryopreservation. Compared to controls, *BRCA1*, but not *BRCA2*, mutation positive women produced lower numbers of eggs (7.4 *vs* 12.4) and had very high chances of low response to ovarian stimulation. Therefore, authors suggested that *BRCA1* mutations are associated with occult primary ovarian insufficiency. Finally, a study by Shapira et al. shows no evidence of an association between *BRCA1/2* mutations with a lower ovarian response in IVF treatment (83).

## Interactions Between BRCA1 and Stress Hormones

The stress hormone hydrocortisone (cortisol) was shown to repress *BRCA1* gene expression in a mouse mammary cell line (84). Furthermore, hydrocortisone was also demonstrated to inhibit the stimulatory effect of estrogen on *BRCA1* expression, hence interfering with estrogen-related signaling in mammary epithelial cells. Hence, down-regulation of BRCA1 by cortisol may constitute a distinct pathological mechanism for involvement of stress hormones in breast carcinogenesis.

In addition, studies have identified a direct role for the unliganded glucocorticoid receptor (GR) in BRCA1 upregulation in the absence of hydrocortisone (85). GR was shown to physically interact with the *BRCA1* gene promoter in the absence of hydrocortisone, whereas the positive effect of GR was lost upon addition of the ligand. Given the fact that low levels of BRCA1 have been correlated with the initiation and progression of sporadic breast cancer, this molecular mechanism may explain the finding that prolonged stress signaling increases breast cancer risk.

## Conclusions

Studies summarized in the present review article emphasize the emerging role of BRCA1 as an important player in metabolic and endocrine regulation. While BRCA1 was discovered by virtue of its roles in cancer biology and its genomic activities, accumulating evidence indicates that BRCA1 displays a spectrum of actions that do not fall within the classical cancer-related types of action.

Among other physiological activities, BRCA1 was shown to induce the metabolic reprogramming of breast cancer cells with ensuing reversal of the Warburg effect. BRCA1 governs important steps of the lipogenetic pathway and has a key role in energy metabolism. BRCA1 interacts with several hormones, including IGF1, estrogens and androgens, cortisol, etc. In addition, BRCA1 seems to be involved in the process of reproduction.

In conclusion, a better understanding of the complex physical and functional interactions between BRCA1 and hormonal and metabolic pathways will have major basic and translational relevance.

## Author Contributions

The author confirms being the sole contributor of this work and has approved it for publication.

## Conflict of Interest

The author declares that the research was conducted in the absence of any commercial or financial relationships that could be construed as a potential conflict of interest.

## Publisher’s Note

All claims expressed in this article are solely those of the authors and do not necessarily represent those of their affiliated organizations, or those of the publisher, the editors and the reviewers. Any product that may be evaluated in this article, or claim that may be made by its manufacturer, is not guaranteed or endorsed by the publisher.
